# A novel room-temperature formaldehyde gas sensor based on walnut-like WO_3_ modification on Ni–graphene composites

**DOI:** 10.3389/fchem.2022.971859

**Published:** 2022-09-09

**Authors:** Shahid Mehmood, Faheem Ullah Khan, Muhmmad Naeem Shah, Junxian Ma, Yatao Yang, Guijun Li, Wei Xu, Xiaojin Zhao, Wei He, Xiaofang Pan

**Affiliations:** ^1^ College of Electronics and Information Engineering, Shenzhen University, Shenzhen, China; ^2^ Key Labortary of Optoelectronics Devices and System of Ministry of Education and Guangdong Province, College of Physics and Optoelctronics Engineering, Shenzhen University, Shenzhen, China; ^3^ Huazhong University of Science and Technology, Wuhan, China

**Keywords:** WO3-Ni-Gr composite, pn heterojunction-based gas sensors, room-temperature formaldehyde sensor, spill-over effect, long-term stability

## Abstract

Ternary composite with great modulation of electron transfers has attracted a lot of attention from the field of high-performance room-temperature (RT) gas sensing. Herein, walnut-like WO_3_-Ni–graphene ternary composites were successfully synthesized by the hydrothermal method for formaldehyde (HCHO) sensing at RT. The structural and morphological analyses were carried out by scanning electron microscopy (SEM), transmission electron microscopy (TEM), X-ray diffraction (XRD), Raman spectroscopy, and X-ray photoelectron spectroscopy (XPS). SEM and TEM studies confirmed that walnut-like WO_3_ nanostructures with an average size of 53 ± 23 nm were functionalized. The Raman and XPS results revealed that, due to the deformation of the O-W-O lattice, surface oxygen vacancies O_v_ and surface-adsorbed oxygen species O_c_ were present. The gas-sensing measurement shows that the response of the WO_3_-Ni-Gr composite (86.8%) was higher than that of the Ni-Gr composite (22.7%) for 500 ppm HCHO at RT. Gas-sensing enhancement can be attributed to a p-n heterojunction formation between WO_3_ and Ni-Gr, O_c_, spill-over effect of Ni decoration, and a special walnut-like structure. Moreover, long term stability (%R = 61.41 ± 1.66) for 30 days and high selectivity in the presence of other gases against HCHO suggested that the proposed sensor could be an ideal candidate for future commercial HCHO-sensing in a real environment.

## Introduction

The precise detection of volatile organic compounds (VOCs), including formaldehyde (HCHO), ammonia (NH_3_), isopropyl alcohol (IPA), ethanol, acetone, and carbon monoxide (CO) in both indoor and outdoor environments is closely related to environmental evaluation, disease diagnosis, and food quality analysis (Chen et al., 2014; Park et al., 2014; Mu et al., 2015). Out of various VOCs, HCHO, a carcinogenic VOC, is threatening human health as an indoor air pollutant (El Sayed et al., 2016; Wang et al., 2018). Specifically, a low level of 1–3 ppm of HCHO can cause nose and eye irritations while contacting with a higher level of 15 ppm can cause death (El Sayed et al., 2016; Wang et al., 2018). Hence, for human health and environmental protection, an effective monitoring approach for HCHO is greatly needed. In addition, the sensor chip with mature and well-performed HCHO detection in human breath can be used in clinics with the advantage of early stage monitoring of diseases such as nasopharyngeal cancer and leukemia (Hopkinson and Schofield, 2018; Möhner et al., 2019). Therefore, in recent decades, different strategies of sensor fabrication have been employed for the detection of HCHO (Hu J. et al., 2021; Liu et al., 2021; Peng et al., 2022; Sun et al., 2022). Ion chromatography (Lorrain et al., 1981), gas chromatography (Yoo et al., 2021), high-performance liquid chromatography (Zhang et al., 2019), and spectrometry (Mohlmann, 1985) are conventionally used as instrumental techniques for HCHO detection. However, high cost of the spectroscopic method and complex/bulky instrumentation for the chromate-graphic technique limit their real-time and field-monitoring usage (Chung et al., 2013). Shortcomings such as long-time sample preparation, expensive operational cost, and large power consumption with the aforementioned traditional techniques have accelerated the development of portable, rapid, and highly sensitive novel sensors. Therefore, chemiresistive gas sensors (Guan et al., 2020; Khan et al., 2021), due to their simplicity and low cost, have motivated the researcher to use advanced nanotechnology-based gas-sensitive materials with extraordinary detection capabilities of HCHO.

In the last two decades, nanomaterials of metal oxides (MOX), conducting polymers, and carbons tubes have emerged as gas-sensitive materials. Among them, MOX, such as ZnO, TiO_2_, SnO_2_, and WO_3_ (Liu et al., 2017; Chen et al., 2018; Chang et al., 2021), based gas sensors because of their excellent sensing performance, environment friendly nature, long time usage, and low-cost properties have been intensively studied for the detection of various VOCs including HCHO. Among various MOXs, because of its superior chemical stability, WO_3_, a typical n-type semiconductor, is specifically used for HCHO detection (Dong et al., 2020). However, pure WO_3_ exhibits low sensitivity and requires high-operational temperature of 200–500°C (which leads to increased power consumption) (Dong et al., 2020). To improve the performance of WO_3_-based gas sensors, the surface of WO_3_ needs to be functionalized with some metal nanoparticles to meet the pre-requisite of practical applications. Ionescu et al. demonstrated the noble metals such as Au, Pt, Au/Pt, and Fe nanoparticles decorated with WO_3_ detected 5, 10, and 15 ppm HCHO under UV light irradiation (Bouchikhi et al., 2020). Herein, the catalytic oxidation of metal nanoparticles and electronic sensitization of WO_3_ in combination can effectively improve the gas-sensing performance. However, inhomogeneous dispersion of metal nanoparticles leading to a relatively poor utilization rate of the active sites for gas sensing still makes the sensors unable to meet the requirement of extremely low concentration of HCHO (or low temperature) (Yuan et al., 2019).

Recently, graphene (Gr)-based sensor, due to its large specific surface area, good mechanical strength, and high sensitivity to electrical perturbation from gas molecules, has been widely used as promising material in high-performance room-temperature gas sensors (Varghese et al., 2015; Kumar et al., 2019). Gr is a p-type semiconductor in nature (Punginsang et al., 2017), when Gr is exposed to HCHO, the conductivity of Gr is affected by weak Van der Walls interaction with HCHO at room temperature. Weak van der Walls indistinctive gas adsorption on graphene results in poor selectivity and reversibility. Typically, graphene-based sensors are studied in the form of graphene composites, by functionalizing with MOX/metals to achieve improved selectivity and reversibility. For example, vertical graphene with tin oxide nanoparticles for highly sensitive room-temperature HCHO sensing (Bo et al., 2018), WO_3_-rGO porous nanocomposite for NH_3_ detection (Jeevitha et al., 2019), GO decorated by a hollow SnO_2_ nanofiber for HCHO detection (Wang et al., 2017), and GO/Ag nanoparticle composite film for sensitive detection toward traces of formaldehyde (Zhang T. et al., 2017). Synergic interaction between Gr sheets and WO_3_ has been credited to enhance the sensing response of hybrid nanocomposites. Similarly, various reports have been published regarding metal functionalization on WO_3_ for tuning selectivity, improving sensitivity, and low-operating temperature gas-sensing measurements (Jeevitha et al., 2019; Bouchikhi et al., 2020). Although the functionalization of Gr could enhance the feature of low-temperature sensing, the degree of improving sensing response by Gr is still finite. Therefore, to enhance the sensing performance, a novel functional material composite consisting of Gr, noble metal (Ni), and metal oxide (WO_3_) is synthesized in this work. A ternary composite of WO_3_-Ni-Gr thus formed can utilize the Gr-sensing characteristic of low temperature operation, Ni as better oxygen dissociation catalysts, and WO_3_ properties of good response and improved selectivity as a synergistic effect with a better sensing performance material for practical application. However, to date, the HCHO sensor based on a WO_3_, Ni, and Gr ternary composite material (WO_3_-Ni-Gr) has not been reported yet.

In view of the aforementioned survey, the sensing characteristics of WO_3_-Ni-Gr and Ni-Gr composites toward HCHO at room temperature are reported and compared. The Ni-Gr composite is synthesized by a simple and facile hydrothermal method and then WO_3_ nanostructures are decorated on the surface of Ni-Gr using the hydrothermal method. The structural and chemical features of WO_3_-Ni-Gr and Ni-Gr composites are characterized by scanning electron microscopy (SEM), transmission electron microscopy (TEM), high resolution transmission electron microscopy (HRTEM), X-ray diffraction (XRD), Raman spectroscopy, and X-ray photoelectron spectroscopy (XPS). WO_3_-Ni-Gr and Ni-Gr composite-based sensors are then fabricated and their sensing behaviors toward various VOCs are tested by a customized gas-sensing analysis system. Data results displayed that WO_3_-Ni-Gr composites have shown an excellent sensing response toward HCHO (1–500 ppm) at room temperature. Compared to Ni-Gr composites, the sensitivity, selectivity, and sensing speed are improved for WO_3_-Ni-Gr sensors. The influence of WO_3_ decoration on Ni-Gr toward HCHO detection for an enhanced sensing performance is investigated. The spill-over effect of Ni decoration, walnut-like morphology of WO_3_, heterojunction formation between WO_3_ and Ni-Gr and the absorbed oxygen species are the factors attributed for improved sensing performance.

## Materials and methods

### Materials

All chemicals are of analytical reagent grade and used without further treatment. Gr, IPA, DMF, and ethanol were obtained from Shanghai Aladddin Biochemical Technology Co. Ltd., while nickel nitrate (NiNO_3_) and tungsten hexachloride (WCl_6_) were purchased from Sigma Aldrich.

### Preparation of Ni-Gr and WO_3_-Ni-Gr composite

**FIGURE 1:**
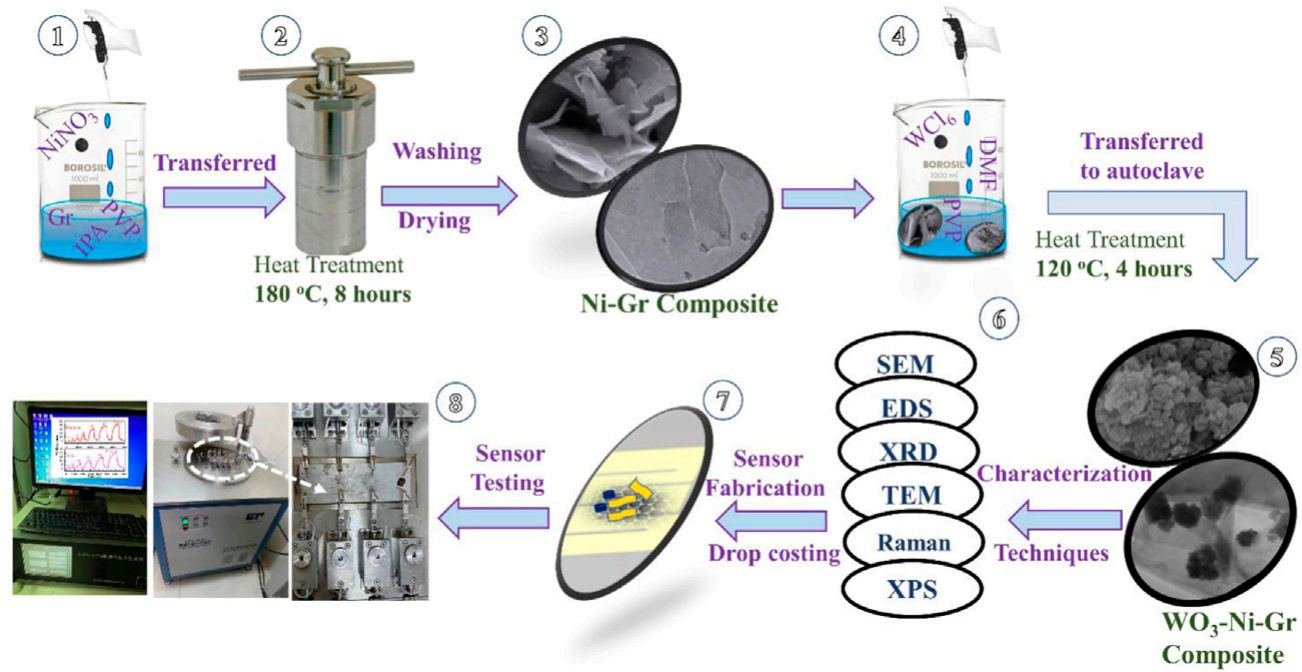
Schematic of the syntheses of Ni-Gr and WO_3_-Ni-Gr composites, sensor fabrication, and testing.

500 mg of Gr nanoplatelets and 125 mg of polyvinylpyrrolidone (PVP) are mixed in 75 ml IPA and the solution is stirred for 1 h. NiNO_3_ (100 mg) is then added into the aforementioned solution and the mixture is stirred vigorously for 1 h. The 100 ml stirred solution is then transferred into Teflon-lined stainless autoclave, heated at 180°C for 8 h in an oven and then cooled at room temperature naturally. Finally, the black precipitates of the Ni-Gr composites are collected by the centrifugation, washed using deionized water several times, and dried at 90°C for 24 h. Steps 1, 2, and 3 in Figure 1 summarize the synthesis procedure of the Ni-Gr composites.

WO_3_-Ni-Gr composites are synthesized by the following procedure: Ni-Gr composites obtained from the first step are separately dissolved in distilled water to form the suspension. 300 mg of WCl_6_ and PVP are dissolved in 30 ml of DMF and then the solution is added slowly in suspension of the Ni-Gr composite. The mixture is stirred continuously for 20 min at room temperature and then transferred into a 50 ml Teflon-lined stainless autoclave and maintained at 120°C for 4 h in an oven and then cooled at room temperature, naturally. The WO_3_-Ni-Gr product is filtered, washed with distilled water and ethanol several times, and dried at 90°C in vacuum over for 24 h. Steps 4 and 5 of Figure 1 illustrate the aforementioned procedure for WO_3_-Ni-Gr composite syntheses.

### Sensor fabrication and testing

10 mg of the Ni-Gr and WO_3_-Ni-Gr composites are separately added into 5 ml deionized water and then ultrasonicated for 20 min to form the homogenous dispersion. Dispersion is then drop-casted (Step 7 of Figure 1) on the electrode to obtain the resistance-type sensor. The sensor is then dried at 180°C for 24 h in the vacuum for better stability. The room-temperature sensing measurement of VOCs (Step 8 of Figure 1) is carried out in an intelligent gas sensing analysis system CGS-4TPs (Beijing Ellite Tech. Co. Ltd.). The main parts of the gas-sensing system include the gas chamber, small probes for the sensor connection, data acquisition board and display, and data storage system (Figure 1 Step 8). Fabricated electrodes are loaded on the sensor platform highlighted by a white circle in Step 8 of Figure 1. Real-time resistance is extracted by the data acquisition board and recorded in the system as resistance. To evaluate the gas-sensing performance, one critical figure of merit is the %response which is defined as:

$$\%R=\left(\frac{R_{g}-R_{a}}{R_{a}}\right)\times 100$$

where R_g_ is the resistance upon exposure of the target gas and R_a_ is the stable resistance of the sensor in air. Furthermore, response time (τ_res_) is the time required to attain 90 % of the saturation resistance as compared to the initial value while recovery time (τ_rec_) is the time required to gain 90 % of the baseline value from the final value. τ_res_ and τ_rec_ are other crucial parameters for gas sensing.

### Characterization detail

Field emission scanning electron microscopy (FESEM) and energy dispersive X-ray spectroscopy (EDS) analyses are carried out using an FEI double-beam scanning electron microscope equipped with an EDS spectrometer for the morphological analysis of Ni-Gr and WO_3_-Ni-Gr composites. XRD analysis is carried out by using the Rigaku-Miniflex-600 Xray diffractometer for structural analysis of both composites. Raman spectroscopy (Thermo DXR2xi) and X-ray photoelectron spectroscopy (XPS) (Thermo Scientific ESCALAB^™^ XI+) are employed to study the vibrational modes and elemental analysis of C, Ni, W, and O, respectively. The room-temperature sensing measurement of VOCs is carried out in an Intelligent Gas Sensing Analysis System CGS-4TPs (Beijing Ellite Tech. Co. Ltd.).

## Result and discussion

**FIGURE 2:**
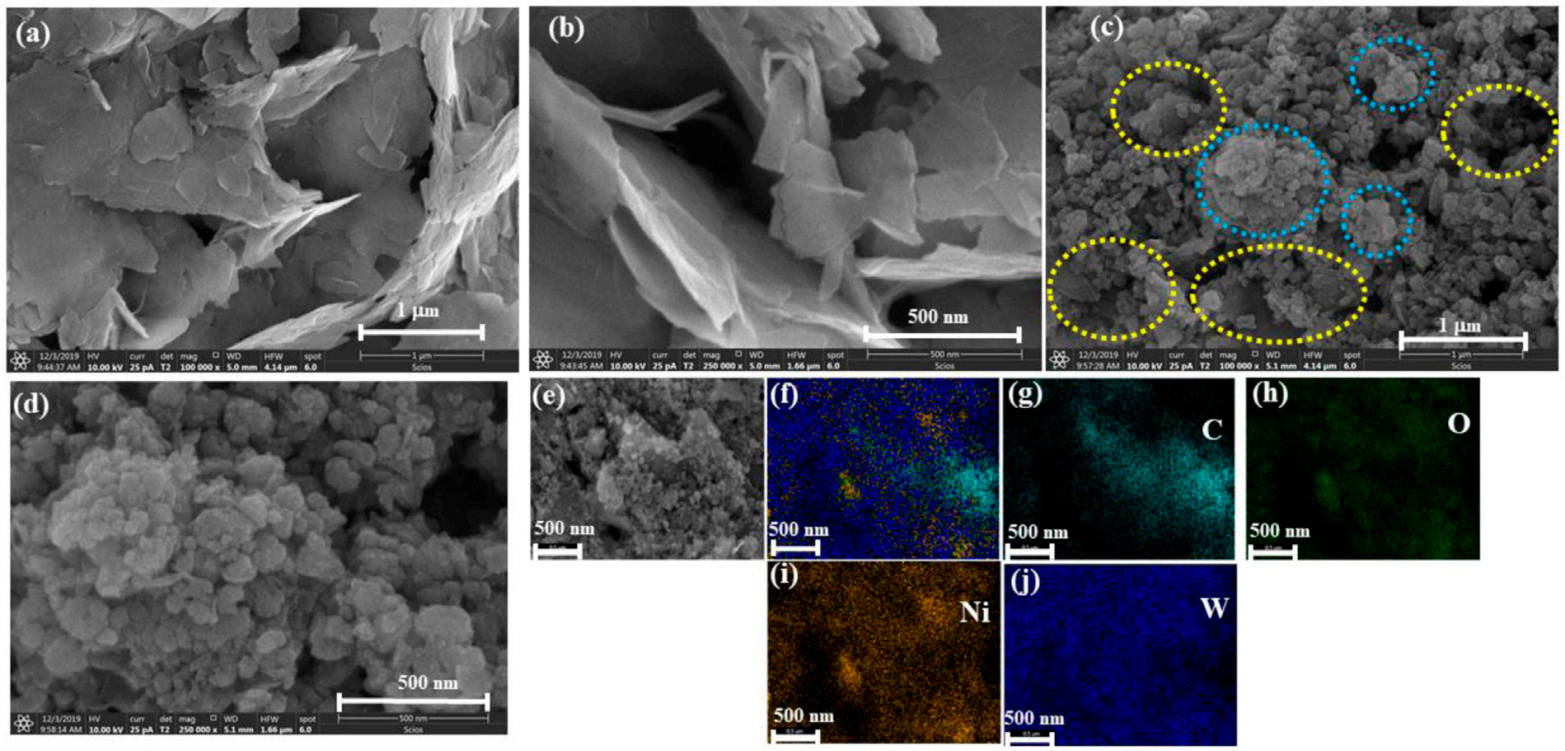
**(A**,**B)** SEM images of Ni-Gr composites. Wrinkled, aggregated graphene sheets are present **(C**,**D)** SEM images of WO_3_-Ni-Gr composites. A bumpy surface approximating the walnut-like surface confirms the attachment of WO_3_ nanostructures. **(E**–**J)** EDS-mapping represents the presence of C, O, Ni, and W.

The morphology of the Ni-Gr composites is analyzed using scanning electron microscopy (SEM). Figures 2A,B show the SEM of the Ni-Gr composite at different resolution. The SEM images of the Ni-Gr composites demonstrate that the wrinkled aggregated graphene sheets are present. Figures 2C,D are SEM images of the WO_3_-Ni-Gr composite at different magnifications. It shows that nanostructures have a bumpy surface resembling a walnut-like surface. Figures 2C,D show that the morphology of the WO_3_-Ni-Gr composite exhibit a substantial change in comparison to Figures 2A,B, and the WO_3_ sheets are intensively decorated on the surface of the graphene, indicating the successful attachment of WO_3_ nanostructures on the surface of Ni-Gr (yellow circles in Figure 2C reveal the crumple graphene structures, while light blue circles represent the WO_3_ structures). This is further proved by EDS and the corresponding elemental mapping of C, O, Ni, and W in Figures 2E–J. The EDS spectrum has shown the presence of C (18 wt %), O (25 wt %), Ni (17 wt %), and W (40 wt %) and this confirms the high purity and stoichiometric nature of the WO_3_-Ni-Gr composite.

**FIGURE 3:**
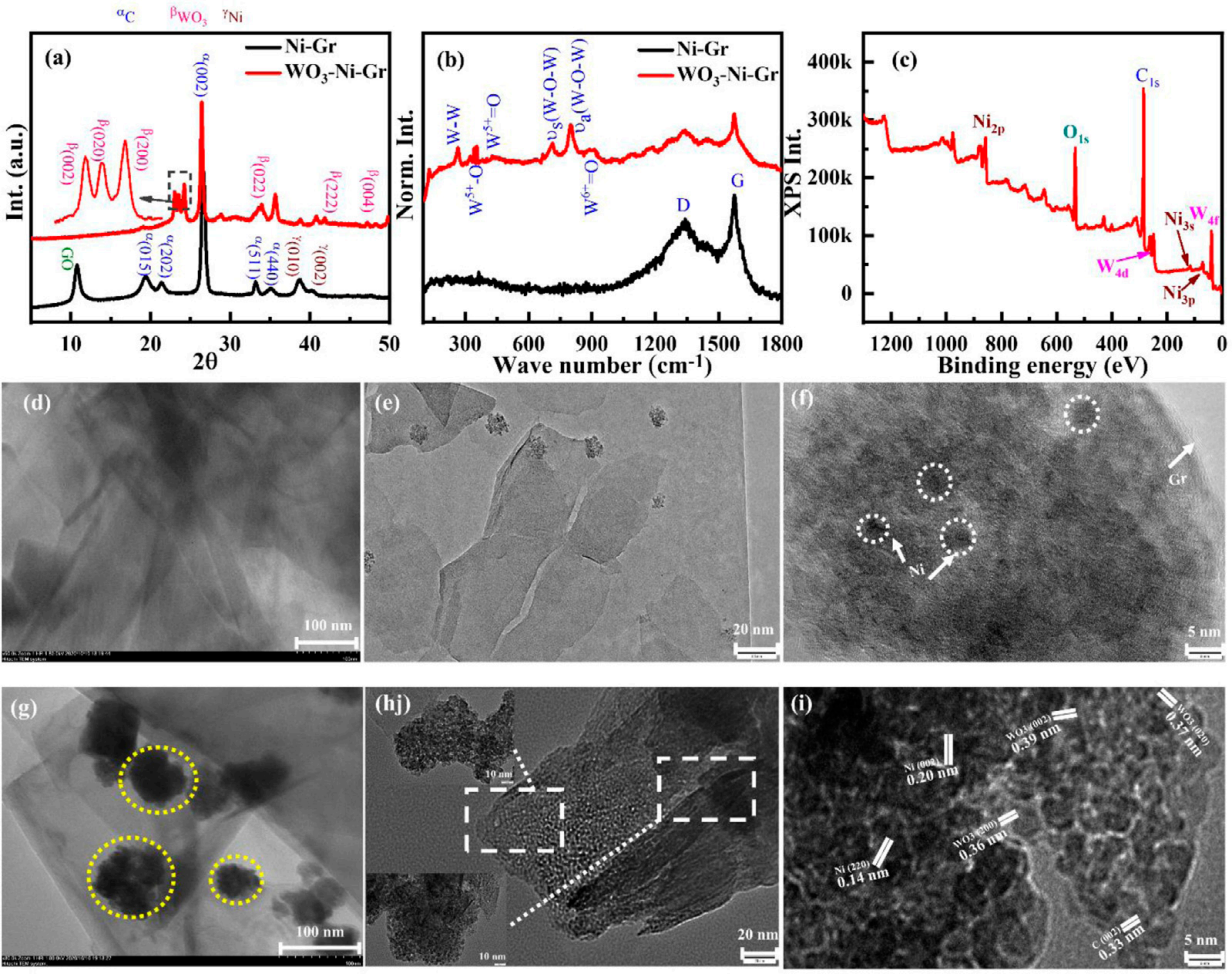
**(A)** XRD patterns of Ni-Gr (bottom) and WO_3_-Ni-Gr (top) composites. Characteristic diffraction peak C (002) at 26.4° represent the graphitic nature of Gr. Minute diffraction peaks of Ni (010) and Ni (002) show that the Ni-Gr composites are formed. 23.0°, 23.5°, and 24.3° which correspond to (002), (020), and (200) reflection of WO_3_ highlighted by the inset confirmed that WO_3_ nanostructures were successfully modified on the Ni-Gr composite. **(B)** The Raman spectra of Ni-Gr (bottom) and WO_3_-Ni-Gr (top) composites. G and D bands were present for Ni-Gr composites. WO_3_ functionalization in the WO_3_-Ni-Gr composite was confirmed by the emergence of new Raman modes of WO_3_ at 240 cm^−1^, 335 cm^−1^, 431 cm^−1^, 805 cm^−1^, and 950 cm^−1^. **(C)** XPS survey scan of the WO_3_-Ni-Gr composite discloses that only W (4f, 4d state), O (1s), C (1s), and Ni (2p, 3s, and 3p) were present. **(D)** TEM images, **(E,F)** HRTEM image of Ni-Gr composites. Layered structures of Gr with dark spots of Ni show the Ni-Gr composite formation. **(G)** TEM images of the WO_3_-Ni-Gr composite. The irregular dark portion highlighted by yellow circles is due to WO_3_ nanostructures with an average size of 53 ± 23 nm. **(H)** HRTEM images of WO_3_-Ni-Gr composites at 20 nm resolution. The inset shows the HRTEM image at 10 nm resolution. **(I)** HRTEM images of WO_3_-Ni-Gr composites at 5 nm resolution. HRTEM images show that crystallographic planes of (002) Gr, (220) and (002) of Ni, and (002), (020), and (200) of WO_3_ are present for the WO_3_-Ni-Gr composite.

The crystal structure of the synthesized composites is studied by X-ray diffraction (XRD), Figure 3A shows the XRD patterns of Ni-Gr and the WO_3_-Ni-Gr composite. The XRD pattern of the Ni-Gr composite (bottom pattern) has presented a strong peak at 26.4° with minute diffraction peaks at 10.7°, 19.4°, 21.8°, 32.8°, 35.8°, 39.1°, and 41.5°. The strong peak at 26.4° corresponds to the (002) diffraction peak of C (Gr), which represents the graphitic nature with a uniform structure of different layer spacing (0.33 nm) (Ansari et al., 2019). The diffraction peak at 10.7° is the characteristic graphene oxide (GO) peak which indicates that the surface of the graphene is functionalized with oxygen or carboxylic (-COOH) species (Tang et al., 2014). The peaks at 19.4°, 21.8°, 32.8°, and 35.8° are attributed to (015), (202), (511), and (440) phase of the C. The diffraction peaks at 39.1° and 41.5° are related to (010) and (002) planes of Ni. The XRD patterns of Ni-Gr have shown the characteristic diffraction peaks of both Gr and Ni which confirm the Ni-Gr composite formation. The XRD pattern of the WO_3_-Ni-Gr composite (red-marked top pattern) demonstrates that new peaks have emerged at 2θ angles of 23.0°, 23.5°, 24.3°, 34.5°, 42.0°, and 47.2°. Peaks at the 2θ angle such as 23.0°, 23.5°, and 24.3° which correspond to (002), (020), and (200) reflections of WO_3_, respectively (Ansari et al., 2019), have been highlighted by the inset in Figure 3A. Other diffraction peaks at the 2θ angle such as 34.5°, 42.0°, and 47.2° are due to (022), (222), and (004) planes of WO_3_, respectively (Ansari et al., 2019). The diffraction peaks of the WO_3_ nanostructures are well matched with JCPDS#20–1324 showing that the orthorhombic phase of WO_3_ is synthesized. The emergence of new peaks for WO_3_ in comparison to the Ni-Gr composites prove the existence of WO_3_ nanostructures on the surface of Ni-Gr composites and thus the successful formation of the WO_3_-Ni-Gr composite.

Raman spectroscopy is conducted to have deeper insight into the structural and electronic characteristics of composites. Figure 3B displays the Raman spectra of both Ni-Gr and WO_3_-Ni-Gr composites. The Raman spectra of the Ni-Gr composite (bottom one) have shown the Raman mode at 1,345 cm^−1^ (D-band) and 1,578 cm^−1^ (G-band) (Basta et al., 2022). G-band is the characteristics of graphitic nature and it is due to C-C vibrations of sp^2^ hybridized carbons while D band is a disorder band which arises from disorder-induced vibration in sp^3^ hybridized carbon (Yang et al., 2021). The presence of D-band can be associated to defect states in Ni-Gr composites due to Ni and COOH functionalizations which are already confirmed in the XRD and TEM spectra. The Raman spectra of the WO_3_-Ni-Gr composite (top spectra in Figure 3B) show that new bands have emerged at wave numbers such as 950 cm^−1^, 801 cm^−1^, and 718 cm^−1^ while in the lower wave number range, 240 cm^−1^, 335 cm^−1^, and 431 cm^−1^; however, G and D bands are at 1,578 cm^−1^ and 1,345 cm^−1^, respectively. New bands at 950 cm^−1^, 801 cm^−1^, and 718 cm^−1^ are due to W^6+^=O, antisymmetric W-O-W and symmetric W-O-W vibrations, respectively (Ozceri et al., 2021) while 240 cm^−1^ (W-W vibration), 335 cm^−1^(W^5+^-O vibration), and 431 cm^−1^ (W^5+^=O vibration) modes are related to deformation lattice O-W-O modes due to oxygen vacancies (Ansari et al., 2019; Fan et al., 2020; Tran et al., 2021). In addition, the G band in the WO_3_-Ni-Gr composite is broadened in comparison to Ni-Gr composites. Occurrence of new bands at 801 cm^−1^ and 718 cm^−1^ and 132–326 cm^−1^ ranges along with the broadening of the G-band can be used as a confirmation that WO_3_ nanostructures are bounded on the surface of Ni-Gr. X-ray photoelectron spectroscopy (XPS) which is an effective technique to verify the surface state of nanomaterials by looking into elemental composition and chemical states is further used. Figure 3C demonstrates the XPS survey scan of WO_3_-Ni-Gr composites, from Figure 3C it is revealed that only W (4f, 4d state), O (1s), C (1s), and Ni (2p, 3s and 3p) are present. The co-existence of the valance states of W, O, C, and Ni without any other impurities can be used as a signature that WO_3_-Ni-Gr composites are successfully synthesized. This is in accordance with the EDS spectrum (Figures 2E–J) where a similar observation of co-existence of W, O, C and Ni is found for WO_3_-Ni-Gr composites.

Transmission electron microscopy (TEM) is also utilized to comprehend the detailed structural and morphological analysis of Ni-Gr and WO_3_-Ni-Gr composites. Figure 3D shows the TEM images of the Ni-Gr composite, where layered structures with dark spots are observed. The layered structures are of graphene while the dark spots are due to presence of Ni. High resolution transmission electron microscopy (HRTEM) of Ni-Gr composites is displayed in Figures 3E,F at the 20 and 5 nm scales, respectively. HRTEM images of Figure 3F demonstrate that the layered structures of Gr are present and the dark spots are due to Ni. Figure 3F shows that the Ni content is dispersed on the surface of Gr. Figure 3G shows the TEM images of WO_3_-Ni-Gr composites, the irregular dark portion signifies that the walnut-like WO_3_ nanostructures are dispersed on the surface of layered Ni-Gr (lighter part). The average size of the WO_3_ nanostructures measured from Figure 3G is 53 ± 23 nm. HRTEM images of WO_3_-Ni-Gr composites at 20 and 5 nm scales are shown in Figures 3H,I. The morphology of Figure 3G is totally different from Figure 3E, which confirmed that WO_3_ nanostructures are decorated on the surface of Ni-Gr. Figure 3I shows the HRTEM image of the WO_3_-Ni-Gr composite indicating that lattice fringes with d-spacings of 0.33, 0.37, 0.36, 0.39, 0.14, and 0.20 nm are present. Lattice fringes with a d-spacing of 0.33 nm are for the (002) plane of Gr (Ansari et al., 2019), while 0.14 and 0.20 nm are for the (220) and (002) planes of Ni, respectively (Jia et al., 2021). 0.39, 0.37, and 0.36 nm correspond to (002), (020), and (200) planes of WO_3_, respectively (Ansari et al., 2019). The crystallographic planes of (002) Gr, (220) and (002) of Ni, and (002), (020), and (200) of WO_3_ confirmed that the WO_3_-Ni-Gr composite is formed.

**FIGURE 4:**
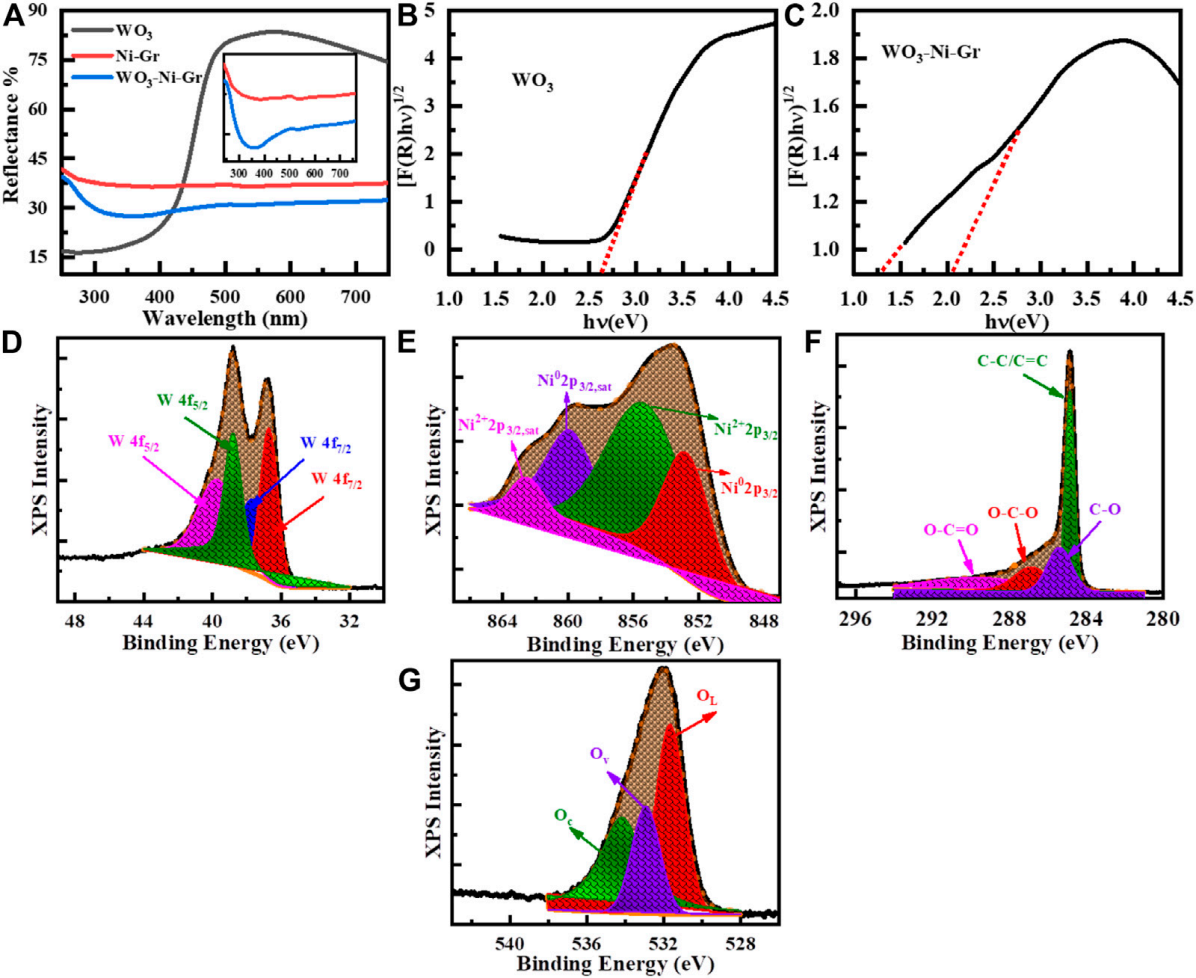
**(A)** UV-Vis diffuse reflectance spectra of WO_3_, Ni-Gr, and WO_3_-Ni-Gr composites. **(B)** Corresponding Kubelka–Munk plot of WO_3_ **(C)** the WO_3_-Ni-Gr composite for band gap estimation. **(D**–**G)** High-resolution XPS spectra of **(D)** W (4f), **(E)** Ni (3p3/2), **(F)** C (1s), and **(G)** O (1s).

UV–Vis diffuse reflectance measurements are employed for the optical property determination of the as-synthesized WO_3_, Ni-Gr composite, and WO_3_-Ni-Gr composite. Figure 4A shows the percentage (%) reflectance as a function of the wavelength (250–750 nm) for WO_3_, Ni-Gr, and WO_3_-Ni-Gr. The absorption edge is founded at about 450 nm for as**-**synthesized WO_3_ nanometers which agrees well with the reported value (Ng et al., 2018). In case of the Ni-Gr composite, no absorption edge is observed. However, for WO_3_-Ni-Gr, an absorption edge is spotted close to 450 nm, as shown in the inset of Figure 4A. The emergence of the absorption edge at about 450 nm in the WO_3_-Ni-Gr composite in comparison to the Ni-Gr composite verified that the WO_3_ nanostructures are successfully modified on Ni-Gr. Kubelka–Munk transformation is then used to obtain the Tauc diagram for the band gap analysis of WO_3_ and the WO_3_-Ni-Gr composite and is shown in Figures 4B,C. From the Kubelka–Munk plots (Figure 4B), the band gap of as-synthesized WO_3_ nanostructures is estimated to be 2.66 eV, consistent with the reported value (Hao et al., 2017). Interestingly, the WO_3_-Ni-Gr composite exhibited two absorption edges at 1.38 and 2.10°eV, respectively (Figure 4C), which can be assigned to GO and WO_3_, respectively (Mohamed, 2019). The speculated value of the band gap of WO_3_ in the WO_3_-Ni-Gr composite is less than the value of pure WO_3_ material. This signifies the enhanced interaction among the ternary composites of WO_3_, Ni, and Gr which can lead to an efficient carrier separation and transformation, and consequently, a junction formation between WO_3_ and Ni-Gr. High-carrier density is observed for the WO_3_-Ni-Gr composite supporting information (Supplementary Figures S1A–D and Supplementary Table S1). The enhanced carrier density for the ternary composite of WO_3_-Ni-Gr is probably due to enhanced charge generation and efficient charge transfer and thus, the junction formation between WO_3_ and Ni-Gr.

Surface states analyses of W, O, C, and Ni are further performed by high-resolution XPS and is given in Figures 4D–G. XPS peak fit 4.1 software is used to find the exact contribution of each valence state and all the fittings of high-resolution XPS are shown in Figures 4D–G. Specifically, the high-resolution XPS spectrum of W 4f doublet is shown in Figure 4D; three peaks are observed at the binding energy of 41, 36.8, and 35.6 eV corresponding to 5p_3/2_, 4f_5/2_, and 4f_7/2_ states of W in WO_3_. The peaks at 36.8 and 35.6 eV in deconvolution peaks of the doublet displays the W^6+^ and W^5+^ oxidation states of W (Fan et al., 2020; Tran et al., 2021). These findings are consistent with the RAMAN results (described in previous section) where the Raman modes of W^5+^=O and W^6+^=O appeared due to the presence of oxygen vacancies. The presence of the 6^+^ oxidation state of W confirms that WO_3_ nanostructures have a covered surface of Ni-Gr composite and the 5^+^ oxidation states reveal that oxygen vacancies are also present. Decomposition of high-resolution spectra of Ni 2p_3/2_, shown in Figure 4E, reveals that four distinct peaks are at a binding energy of 863.0, 859.4, 855.4, and 862.5 eV. The peak at 852.8 eV corresponds to the metallic form of Ni (3p3/2) with its satellite peak Ni (3p_3/2, sat_) at 859.4 eV (Darvishi et al., 2017; Ren et al., 2020). Similarly, the peak at 855.4 eV shows nickel hydroxide (Ni^2+^ oxidation state, 3p_3/2_) with its satellite peak (3p_3/2, sat_) at 862.5 eV (Darvishi et al., 2017; Ren et al., 2020). This indicates that Ni^0^ and Ni^2+^ states are present on the surface of the WO_3_-Ni-Gr composite which can act as better oxygen dissociation catalysts. This dissociation results in the rapid diffusion of the gas molecules to the surface vacancies for enhanced sensitivity. The high-resolution spectra of C (1s) peak for the WO_3_-Ni-Gr composite is shown in Figure 4F; the four peaks are at 288.7, 287.1, 285.6, and 284.5 eV. The main peak at 284.5 eV arises due to C-C/C=C bonding while peaks at 288.7, 287.1, and 285.6 eV are attributed to O-C=O, O-C-O, and C-O bonds containing oxygen, respectively (Fan et al., 2020; Wei et al., 2021). The presence of O-C=O, O-C-O, and C-O bonds can be related to the presence of abundant oxygen species on the surface of WO_3_-Ni-Gr composites. The deconvoluted three peak spectra of O of the composite are shown in Figure 4G; the peaks are observed at 534.2, 532.9, and 531.6 eV. The peak at 534.2 eV is due to surface-chemisorbed oxygen species (Oc), the second peak at 532.9 eV could be attributed to oxygen vacancies (O_v_), and the third peak at 531.8 eV has been ascribed to lattice oxygen species (O_L_) (Fan et al., 2020; Wei et al., 2021). The O_v_ peak represents the bonding of WO_3_ nanostructures at the surface of Ni-Gr, while O_v_ and O_c_ signify that oxygen vacancies and adsorbed oxygen species are on the WO_3_-Ni-Gr composite. It is reported by various groups that the presence of O_v_ and O_c_ on the material surface is related to active adsorption sites, which can enhance the sensitivity of the material toward gas sensing.

### Sensing properties of sensors

**FIGURE 5:**
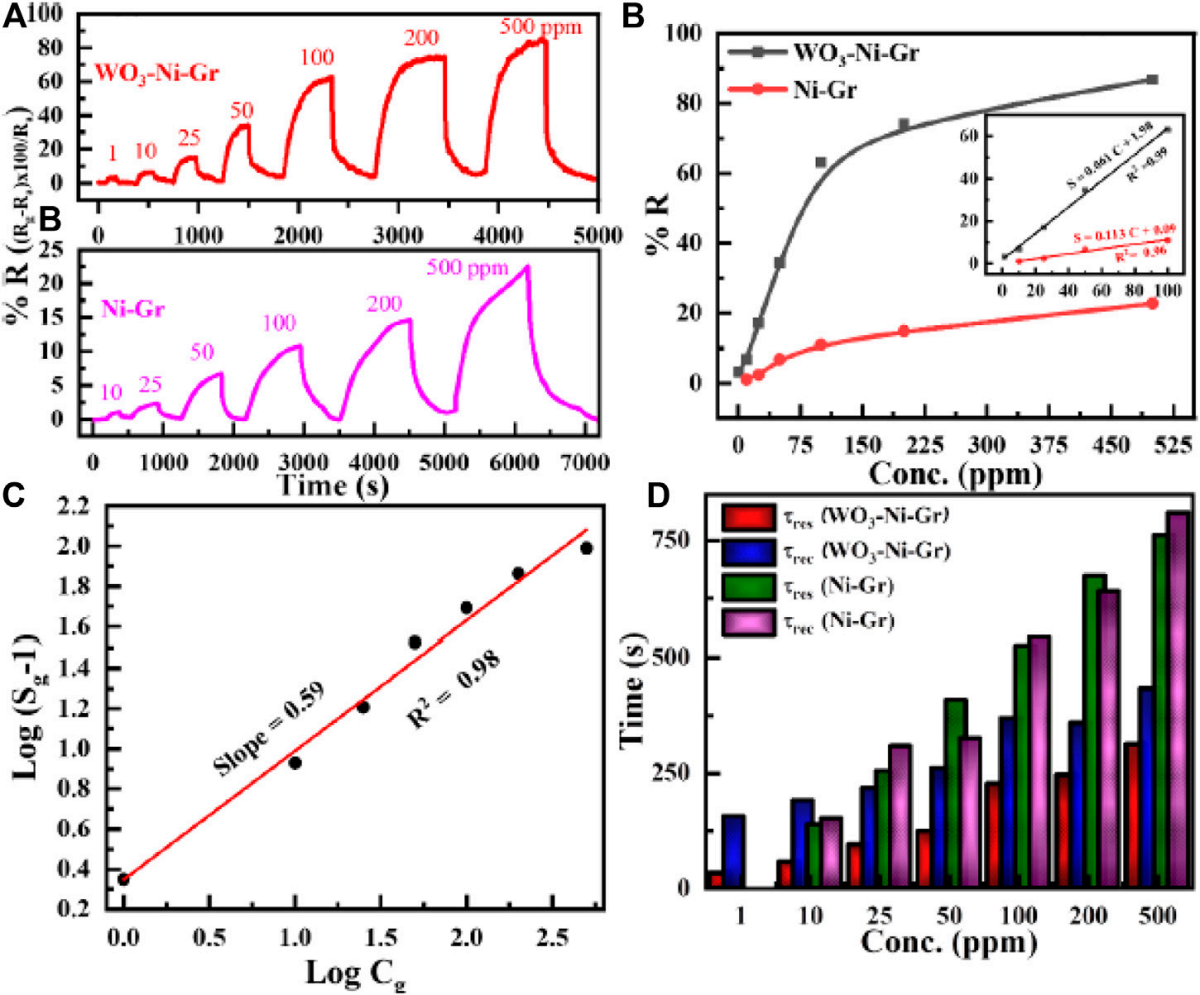
**(A)** Transient responses toward varying concentrations of formaldehyde (HCHO), 10–500 ppm for Ni-Gr (bottom), and 1–500 ppm for the WO_3_-Ni-Gr composite (top) at room temperature. **(B)** The relationship between the response and the concentration (up to 500 ppm) for both sensors against HCHO. Inset of Figure **(B)** shows the linear trend for both composites for 1 ≤ C ≤ 100), where C is the concentration in ppm. **(C)** Logarithm of % response $\left(S_{g}-1\right)$ as a function of the logarithm of HCHO concentration in ppm. The linear relationship with a slope of 0.59 shows that the absorbed oxygen species on the surface of WO_3_-Ni-Gr are mainly $O_{2}^{-}$ species. **(D)** t_res_ and t_rec_ for both WO_3_-Ni-Gr and Ni-Gr sensors are plotted as a function HCHO concentration (ppm). The fast sensing speed of the WO_3_-Ni-Gr based sensor toward HCHO is observed in comparison to the Ni-Gr sensor.

To evaluate the sensing properties of the WO_3_-Ni-Gr and Ni-Gr sensors toward a range of HCHO concentrations, dynamic response measurements are performed. Thus, Figure 5A shows the dynamic response/recovery transient curves of the WO_3_-Ni-Gr- and Ni-Gr-based sensors against various concentrations of HCHO (10–500 ppm) at room temperature. As expected, the gradual increase in resistance of each sensor is observed when increasing the HCHO concentration, with a drop of resistance to the baseline when HCHO is removed from the system. Apparently, the WO_3_-Ni-Gr-based sensor possesses a higher response in comparison to the Ni-Gr sensor, and the response value to 500 ppm of HCHO is 86.8 for the WO_3_-Ni-Gr sensor while the response is only 22.7 for the Ni-Gr sensor. More importantly, for the Ni-Gr sensor, no sensing response is observed for 1 ppm and the detection limit of the WO_3_-Ni-Gr sensor is 1 ppm while for Ni-Gr, it is 10 ppm. Furthermore, the sensing performance of both sensors is evaluated by plotting the sensing response as a function of concentration (ppm) and is displayed in Figure 5B. The response for the WO_3_-Ni-Gr sensor is 3.24, 6.76, 17.1, 34.5, 62.8, 74.1, and 86.8 against 1, 10, 25, 50, 100, 200, and 500 ppm, respectively. Similarly, the responses for the Ni-Gr sensor are 1.11, 2.38, 6.77, 11.04, 14.83, and 22.7 against 10, 25, 50, 100, 200, and 500 ppm, respectively. With the increase in HCHO concentration, the response is increased linearly for both sensors in the range of 1–100 ppm. When the concentration is 100 ppm or above, the response for both sensors increases slowly, which demonstrated the saturation for a higher concentration of HCHO. In the range of 1–100 ppm for both sensors, more active sites are available on the material surface for gas adsorption which results in a rapid increase in the response and larger slope. However, for concentrations >100 ppm, active surface sites available for gas adsorption on the material surface are less, gas adsorption will be saturated, increase in response will gradually reduce, and the slope will be diminishing. It is worth noting that, for all HCHO concentration ranges (1–500 ppm), the response of WO_3_-Ni-Gr is always greater than the Ni- Gr sensors. Inset of Figure 5B reveals that the response curve vs. HCHO concentration shows an almost linear trend for the WO_3_-Ni-Gr and Ni-Gr sensors. The approximation formula used for the linear regression analysis: *S* = 0.061 *C* + 1.98 (1 ≤ *C ≤* 100) for WO_3_-Ni-Gr and *S* = 0.113 *C* + 0.09 (1 ≤ *C ≤* 100) for Ni-Gr, where *S* and *C* are the % responses of the sensor and the concentration of HCHO, respectively. The coefficient of determination *R* ^2^ for WO_3_-Ni-Gr and Ni-Gr is 0.99 and 0.96, respectively. The gas detection limit of HCHO for the WO_3_-Ni-Gr sensor is 1 ppm with a sensing response of 3.24 while that of Ni-Gr sensors is 10 ppm with a response of 1.11. Thus, the improved sensing response characteristics (86.8) and the 1 ppm detection limit in this present study indicate the promising future application of the WO_3_-Ni-Gr sensor for HCHO detection.

WO_3_-Ni-Gr sensors demonstrated good sensing abilities toward HCHO over the concentration range 1–100 ppm. The gas (HCHO) adsorption on the surface of semiconducting oxides such as WO_3_ can be expressed empirically by [39]:

$$S_{g}=1+aC_{g}^{b}$$ (1)

where in Eq. 1, Sg (% response) is sensitivity, C_g_ is the HCHO concentration, and b, a are constants depending upon the sensor material and gas sensor type. The value of constant b can be either 1 or 0.5, and it is related to type of the surface interaction between the HCHO molecule and the chemisorbed oxygen species. The value of b is 1 for $O^{-}$ oxygen species while 0.5 for $O_{2}^{-}$ oxygen species (Kaneti et al., 2013). Eq. 1 can also be written as

$$\log\left(S_{g}-1\right)=\text{loga}+b\log\left(C_{g}\right)$$ (2)

According to Eq. 2, the relation between $\log\left(S_{g}-1\right)$ and $\text{log }\left(C_{g}\right)$ is linear and from the slope of $\log\left(S_{g}-1\right)$ and $\text{log }\left(C_{g}\right)$ plot is b. Figure 5C represents the logarithm of % response $\left(S_{g}-1\right)$ as a function of the logarithm of HCHO concentration in ppm. It is clear from Figure 5C that a linear relationship is observed between the logarithm of % response and logarithm of concentration. The slope (b) is 0.59, which is relatively close to 0.5, suggesting that the absorbed oxygen species on the surface of WO_3_-Ni-Gr are mainly $O_{2}^{-}$ species.

The sensing speed of WO_3_-Ni-Gr and Ni-Gr sensors toward HCHO is estimated by the common practice of response time (τ_res_) and recovery time (τ_res_). τ_res_ is defined as the time required by the sensor to reach 90 % of maximum resistance from baseline resistance when exposed to HCHO gas, while τ_rec_ is the time elapsed by the sensor to recover 90 % of baseline resistance when restored to air. τ_res_ and τ_rec,_ for both WO_3_-Ni-Gr and Ni-Gr sensors as a function increasing HCHO concentration (ppm), are displayed in Figure 5D. τ_res_/τ_rec_ for WO_3_-Ni-Gr are 32s/156s, 56s/190s, 94s/217s, 123/200, 226/369, 245/358, and 312/433 s when exposed to HCHO at 1, 10, 25, 50, 100, 200, and 500 ppm, respectively. In addition, τ_res_/τ_rec_ for Ni-Gr are 138s/151 s, 254s/308 s, 408s/325 s, 524s/541 s, 673s/640 s, and 761s/840 s at 1, 10, 25, 50, 100, 200, and 500 ppm, respectively. Apparently, the sensing speed of the WO_3_-Ni-Gr based sensor is fast for all concentrations in comparison to the Ni-Gr sensor. τ_res_ is 2.4, 2.7, 3.31, 2.31, 2.74, and 2.29 factor low for 10, 25, 50, 100, 200, and 500 ppm, respectively of HCHO. Similarly, τ_rec_ is 0.79, 1.41, 1.62, 1.46, 1.78, and 1.93 factor low for 10, 25, 50, 100, 200, and 500 ppm, respectively of HCHO. Smaller values of τ_res/_τ_rec_ for the WO_3_-Ni-Gr sensor demonstrate that WO_3_ loading at the Ni-Gr sensor has promoted the catalytic activity by rapid dissociation of oxygen molecules. The rapid dissociation of oxygen molecules by both Ni and WO_3_ has resulted in a fast diffusion and adsorption of ions-absorbed oxygen on the surface of WO_3_-Ni-Gr. τ_res/_τ_rec_ of both the sensors tends to increase gradually with HCHO concentration. This is because, adsorption has been slowed for higher density of HCHO and takes a longer time for adsorption of HCHO gas; on the other hand, higher density of HCHO adsorbed on the surface of both sensors has prolonged the desorption (He et al., 2019).

**FIGURE 6:**
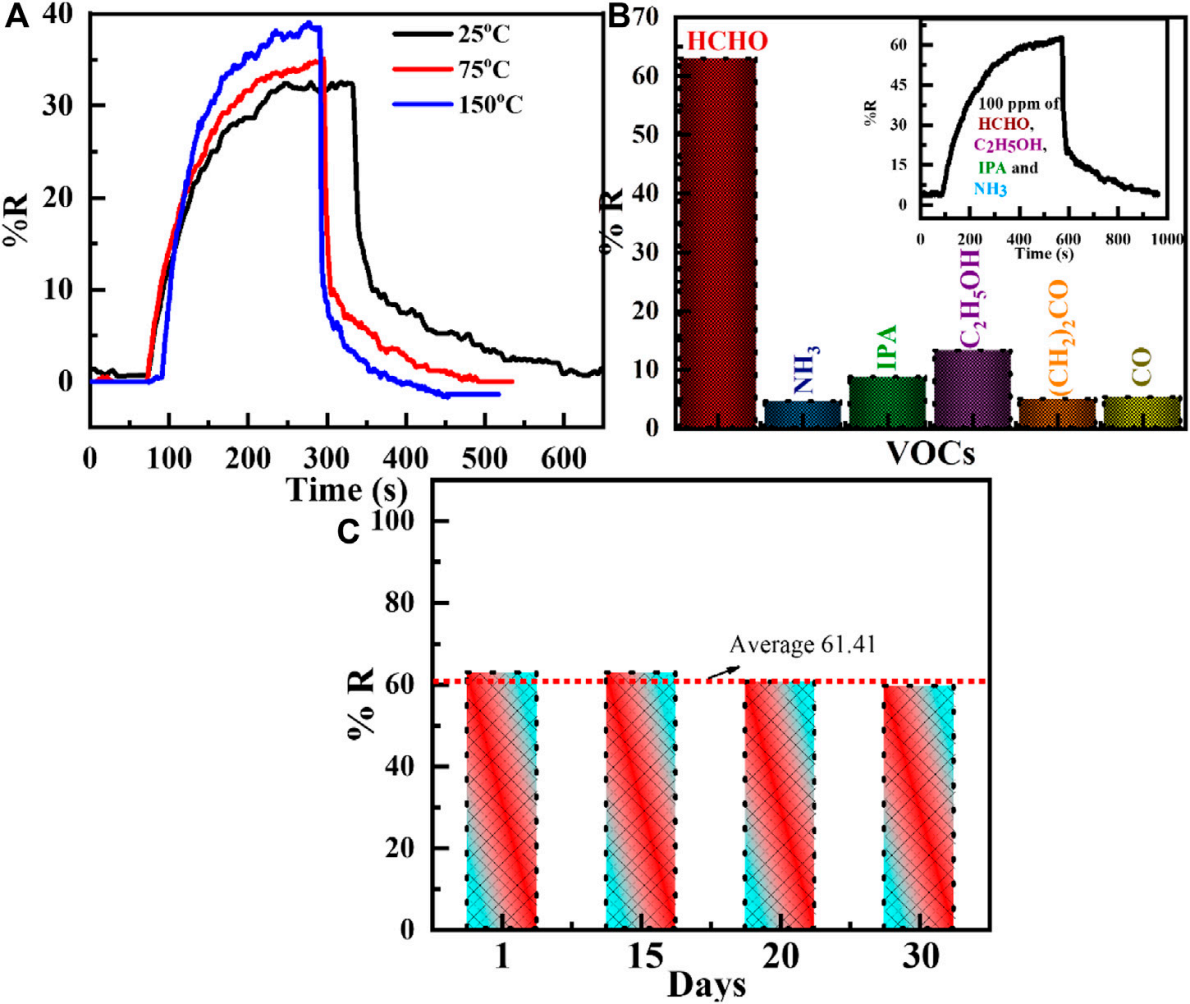
**(A)** Transient responses of the WO_3_-Ni-Gr composite toward 50 ppm of formaldehyde (HCHO) for different temperatures such as 25°C, 75°C, and 150°C. The response of the sensor to HCHO gas increases as the temperature increases. **(B)** Histogram of the sensing response of WO_3_-Ni-Gr composites against various VOCs such formaldehyde, NH_3_, IPA, ethanol, acetone, and CO. Inset of Figure **(B)** shows the selectivity of theWO_3_-Ni-Gr composite toward 100 ppm HCHO in the presence 100 ppm of other interfering gases such as C_2_H_5_OH, IPA, and NH_3_. Negligible change in the response value in the presence of other interfering gases was observed. **(C)** The histogram of the sensing response for 100 ppm HCHO at 1st, 15th, 20^th^, and 30th days. It is obvious that the sensor was stable for a long time.

The sensing performance of the WO_3_-Ni- Gr composite is evaluated for different temperatures toward 50 ppm of HCHO and transient response measurements at 25°C, 75°C, and 150°C, as shown in Figure 6A. From Figure 6A, it is noted that the response of the sensor to HCHO gas increases as the temperature increases. The responses of the WO_3_-Ni- Gr sensor are 32.4, 34.9, and 38.6 at 25°C, 75°C, and 150°C temperature values, respectively. The sensor response is increased to 1.19 times as the temperature is increased to 150°C. Moreover, the response time and recovery time decreases as the temperature is increased. It can be observed that the sensor has the capability to detect HCHO over a wide temperature range, indicating that the WO_3_-Ni-Gr sensor can be used at low and high temperatures for different applications.

The selectivity of the sensor toward specific gases in the presence of other gases is an essential factor for effective practical/commercial use, so, the WO_3_-Ni-Gr sensor is subjected to 100 ppm of similar gases including ammonia (NH_3_), isopropyl alcohol (IPA), ethanol, acetone, and carbon monoxide (CO) at room temperature. The results of the responses for each gas are represented in the histogram in Figure 6B, respectively. The responses of the WO_3_-Ni-Gr sensor are 62.8, 4.5, 8.6, 13.2, 4.9, and 5.2 against 100 ppm of HCHO, NH_3_, IPA, ethanol, acetone, and CO, respectively. The experimental results clearly confirm the higher response of HCHO in comparison to other gases. The inset of Figure 6B shows the dynamic sensing response of 100 ppm HCHO in the presence of 100 ppm of other interfering gases such as C_2_H_5_OH, IPA, and NH_3_. It can be clearly found that the response is 62.1 which is very close to 62.8 of the initial response value of HCHO alone. Negligible change in the response value in the presence of other interfering gases suggested that the specificity of the WO_3_-Ni-Gr sensor toward HCHO is not affected by other gases. This suggested that WO_3_-loaded Ni-Gr sensor can be used as a sensing layer for HCHO detection in real environments.

Furthermore, the validity of the sensor is also established by long term stability, which is a key point for the use of the sensor in commercial applications. The long-term stability is inspected against 100 ppm of HCHO for 30 days at room temperature and is shown in Figure 6C. The responses of the WO_3_-Ni-Gr sensor are 62.81, 62.79, 60.52, and 59.5 for 1, 15, 20, and 30 days, respectively. It is validated from Figure 6C that the sensing responses are highly consistent for 30 days (average ± standard deviation of the response: 61.41 ± 1.66) or 2.74 % change in the response value. These results demonstrated that the WO_3_-Ni-Gr sensor has excellent long-term stability and promising potential when used to detect HCHO vapors. Table 1 presents the comparison between the proposed sensor with other sensors reported in the literature. The comparison results prove that the WO_3_-Ni-Gr sensor reported here features the highest response at room temperature. This indicates that the obtained sensor is the best candidate for HCHO detection.

**TABLE 1:** Responses of various reported HCHO gas sensors.

Sensing material	HCHO Conc. (ppm)	Temp. °C	Min. Conc. (ppm)	*%R*	Ref
Amine-functionalized GO	100	25	50	13	Song et al. (2020)
CuO/rGO hybrids	100	25	2	4	Zhang D. et al. (2017)
RGO/MoS_2_ hybrid films	100	25	2.5	2.75	Li et al. (2017)
Zn doped Ce_2_O_3_	50	32	1	34	Daniel et al. (2020)
RGO/PMMA	300	25	10	12	Chuang et al. (2015)
Present work	1	25	1	3.4	This work
	500	25	1	86.6	This work

**FIGURE 7:**
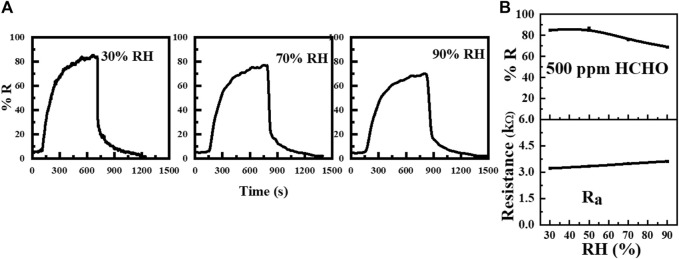
**(A)** Transient responses (% R) of the WO_3_-Ni-Gr sensor to 500 ppm of HCHO at 30 % RH, 70 %, and 90 % RH. **(B)** Plot of responses (% R) and base-line resistance as a function of RH.

To further evaluate the sensing performance of the WO_3_-Ni-Gr sample against humidity, the dynamic response curves to 500 ppm of HCHO are shown separately at 70 % RH and 90 % RH in Figure 7A and their base line resistances and response (% R) as a function of RH are plotted in Figure 7B. The base line resistances (Ra) are **3.33, 3.33, 3.48,** and **3.68** kΩ at **30, 50, 70** and **90** % RH, respectively. The Response ((%R) against 500 ppm ethanol at **30, 50, 70,** and **90** % RH are 85.1, 86.8, 76.1, and 68.7, respectively from Figure 7B. The response (sensitivity) of the sensor decreases slightly as the humidity increases and the maximum decrease in response was only 20.8%. A slight variation in the response as the humidity increases demonstrated that the humidity has a very slight effect on the performance of the WO_3_-Ni-Gr sensor. Increased resistance under a humid environment can be accounted for the reaction of water vapors with absorbed oxygen species (Hu Q. et al., 2021). However, inherent anti-humidity NiO characteristics (Cheng et al., 2021) are the main factors attributing to the anti-humidity performance of the WO_3_-Ni-Gr sample. NiO plays the role of a humidity absorber, and the residue-less water vapor would compete with the target gas.

### Gas-sensing mechanism

The general operational sensing mechanism for resistance-type sensors is based on surface adsorption which results in a change in the carrier concentration and the resistance of the material. Oxygen molecules absorbed on the surface of the material forms ionized chemisorbed oxygen species (O^2−^, O^−^ or $O_{2}^{-}$ ), and the high electronegative chemisorbed oxygen species facilitate the capture of free electrons from the sensing material. For the Ni-Gr sensor proposed in this presented work, the depletion of negative charges on the adsorption of ionized oxygen species in Gr gives rise to an induced electric dipole, which converts the graphene sheets from a gapless semiconductor to a p-type material (Punginsang et al., 2017). Ni in the Ni-Gr composite, due to their metallic nature on the surface of graphene, acts as better oxygen catalysts that fasten the diffusion of the vapor molecules to the surface vacancies. A stronger p-type character is introduced when the Ni metal is introduced on the surface of Gr because the value of the work function of Gr^+^-adsorbed oxygen species (Garg et al., 2014) is less than that of Ni^+^-adsorbed oxygen species (Raaen and Hunvik, 2020). When Ni is brought in contact with Gr, electrons flow from Gr^+^-adsorbed oxygen species to Ni^+^-adsorbed oxygen species until the Fermi level is aligned. This depletion of electrons from Gr on interaction with Ni increases the hole carrier in Gr and hence the p-type behavior. Ni decoration on the surface of Gr will form an extra depletion region by producing more active sites for oxygen adsorption and will enhance the migration of oxygen molecules on the Gr surface. Because of this spill-over effect (Sun et al., 2013), more adsorption of oxygen species will occur on the surface of Gr, and free electrons will be captured from Gr resulting in chemisorbed ion species. Chemisorbed oxygen species formed on p-type Ni-Gr when exposed in air at room temperature is given by Eq. 3.

$$O_{2}\left(gas\right)\to O_{2}^{-}\left(ads\right)+h_{*}\left(<100\mathrm{{}^{\circ}C}\right)$$ (3)

**FIGURE 8:**
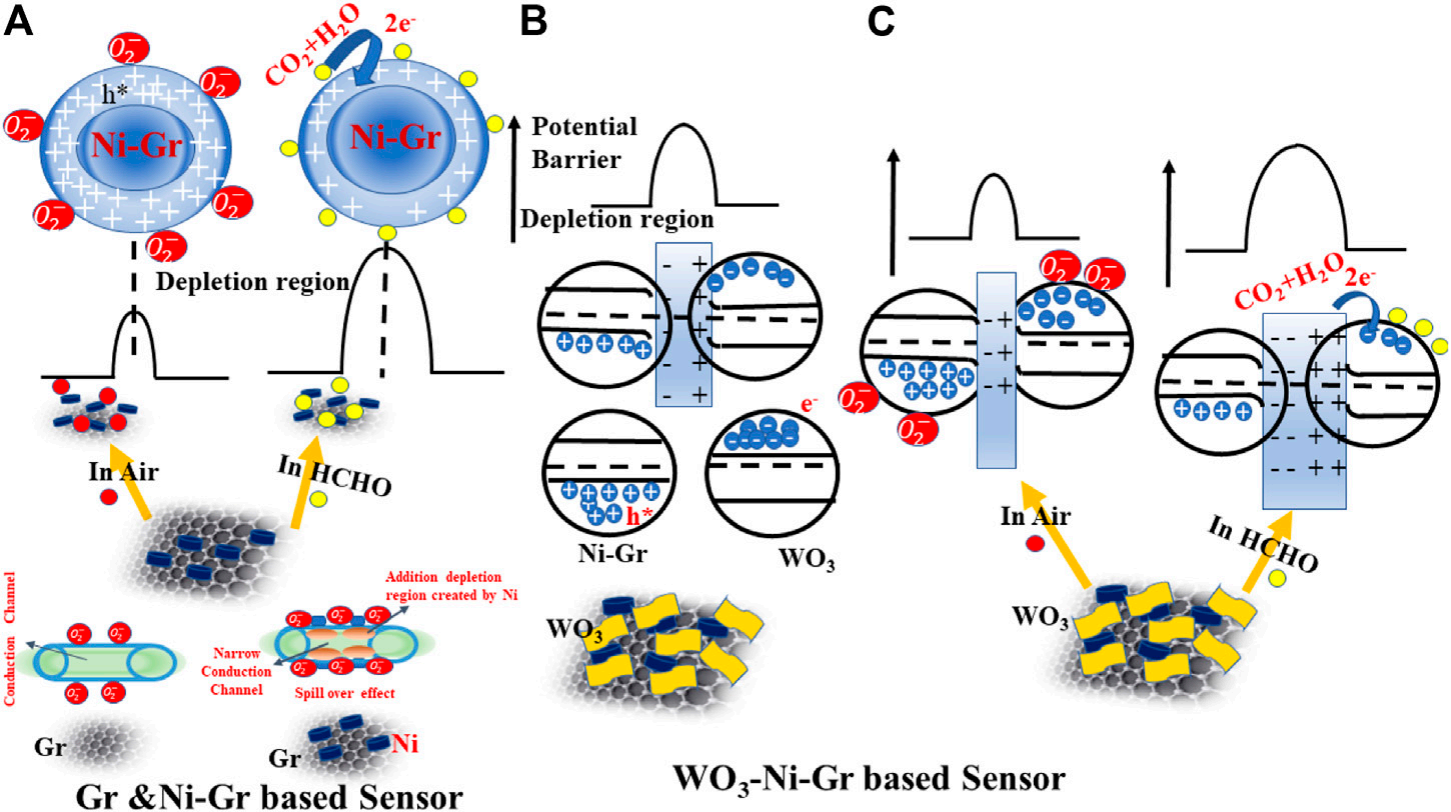
Schematic diagram for the passible sensing mechanism of **(A)** Ni-Gr based sensor. Ni-Gr composites exhibit the p-type behavior. In air-ionized chemisorbed oxygen species, due to the capture of electrons will increase the hole concentration (small resistance), while on HCHO exposure, the captured electrons are released back to the Ni-Gr composite and will decrease the hole concentrations (high resistance). **(B)** A possible mechanism of the WO_3_-Ni-Gr heterojunction formation between WO_3_ and Ni-Gr. Electrons from WO_3_ migrate to Ni-Gr to form the depletion region and heterojunction. **(C)** Schematic of a possible sensing mechanism of the WO_3_-Ni-Gr heterojunction on air and HCHO exposure.

Ionized chemisorbed oxygen species due to electron capture from the material will make the hole concentration increase, the depletion region at the surface thinned, and the resistance decreased (shown in Figure 8A). When the reducing gas HCHO is introduced in the system, holes will be consumed as shown in Eq. 4. This will cause a decrease in hole concentration, widen the depletion region, and hence the high-resistance value (shown in Figure 8A). This is also confirmed in the gas-sensing response of Figure 5A where the resistance of the Ni-Gr sensor is increased on exposure of HCHO.

$$HCHO_{ads}+O_{2}^{-}+h_{*}\to CO_{2}+H_{2}O$$ (4)

The possible chemical reaction described in Eqs 3, 4 at room temperature is reversible and unstable [(Ye et al., 2016)]. When air is flowed again, the aforementioned chemical reaction will be conducted in opposite reactions and $O_{2}^{-}$ is reproduced, and the latter surface attains its initial baseline resistance Ra. In the WO_3_-Ni-Gr sensor, WO_3_ is decorated at the surface of the Ni-Gr sensor (confirmed by HRTEM, EDS, and XPS in earlier sections). As WO_3_ is an n-type material (Dong et al., 2020), a p-n heterojunction between the junction of WO_3_ and Gr will be formed (Jeevitha et al., 2019), contributing to the greatly enhanced HCHO gas-sensing performance, especially long-term stability and high sensitivity for low-concentration HCHO. Specifically, when WO_3_ is exposed in air, chemisorbed oxygen species $O_{2}^{-}$ are formed which is already validated from the previous section that the value of b obtained from the logarithm of % response and logarithm of concentration is 0.59. Moreover, the high-resolution XPS spectra of O (Figure 4G) have verified that oxygen vacancies O_v_ and adsorbed oxygen O_c_ species are on the WO_3_-Ni-Gr composite. For n-type WO_3_, the processes of $O_{2}^{-}$ can be explained by Eq. 5

$$O_{2}\left(gas\right)+e^{-}\to O_{2}^{-}\left(ads\right)\;\left(<100\mathrm{{}^{\circ}C}\right)$$ (5)

Electrons will be captured by chemisorbed oxygen species and hence the depletion region will be widened, and resistance will be increased. However, when it is exposed to HCHO, the captured electrons from the oxygen species will be released back to WO_3_ by Eq. 6. Hence the depletion region will be narrowed, and resistance will be decreased.

$$HCHO_{ads}+O_{2}^{-}\to CO_{2}+H_{2}O+e^{-}$$ (6)

From the aforementioned discussion, free electron dominates the conductivity in WO_3_, and hole dominates the conductivity in Gr. When these two are brought in contact, electrons from WO_3_ tend to migrate to graphene and holes flow from the graphene to WO_3_, due to the difference in their work function until the Fermi levels (EF) from the two sides are aligned, leading to the formation of the p-n heterojunction (shown in Figure 8B). A depletion layer is formed at the interface of both the materials (hole depletion on Gr and electron depletion on WO_3_). The p-type behavior of the WO_3_-Ni-Gr composite is also confirmed in the Mott–Schottky (MS) plot where the negative slope is observed supporting information (Supplementary Figure S1D). When the WO_3_-Ni-Gr sensor is exposed to HCHO, the chemisorbed oxygen species $O_{2}^{-}$ will react with HCHO as given by Eq. 3. WO_3_ and these electrons combine with the holes in Ni-Gr and hence the concentration of holes is further decreased. The migration of electrons to WO_3_ on HCHO absorption takes place and increases the depletion region with the enhanced potential barrier for charge transfer which leads to an increase in gas resistance (schematic of process in Figure 8C). This net change in the holes or charge concentration without and with the HCHO presence contributes to sensor response (Sun et al., 2019). When air is inserted in the chamber, HCHO attached to the sensor surface will be removed and ionized chemisorbed oxygen species will be formed. The ionized chemisorbed oxygen species, due to the capture of electrons from the WO_3_ conduction band, will result in an increased hole concentration in the system, the depletion region will be thinned, and the resistance in air will be reduced (Sun et al., 2019) (schematic represented in Figure 8C). The increased capability of sensing response as the temperature increases is due to the accumulation of more negative charges around ionized oxygen.

For temperature <100°C, chemisorbed oxygen species formed on p-type Ni-Gr when exposed in air at room temperature is given by Eq. 3. However, as the temperature is > 100°C, more electric dipoles are induced as given by Eq. 7. More negative charges are accumulated around ionized oxygen and result in the depletion of negative charges in graphene which give rise to an induced electric dipole.

$$O_{2}\left(gas\right)\to \;2O^{-}\;\left(ads\right)+2h_{*}\left(>100{}^{\circ}C\right)$$ (7)

The induced electric dipole increases the probability of HCHO absorption by ionized oxygen molecules (atoms) and nickel at higher temperatures; consequently, the response and recovery times are reduced. An improved sensing response of 86.8 in the WO_3_-Ni-Gr sensor in comparison to the response of 22.7 in the Ni-Gr sensor is due to the formation of the p-n heterojunction at the interface of WO_3_ and Ni-Gr. Furthermore, an improved gas-sensing response after WO_3_ decoration on Ni-Gr is closely related to the walnut-like morphology (confirmed in SEM (Figures 2C,D) and TEM sections (Figure 3G) of WO_3_. The walnut-like (uneven surface) morphology of WO_3_ provides more active sites for HCHO molecule adsorption which provide more involvement to reactions with adsorbed oxygen species ( $O_{2}^{-}$ ) and hence the sensing response is improved. It is worth noting that the mechanisms for higher selectivity toward HCHO are extremely complex (Wang et al., 2014). One possibility is the lower dissociation bond dissociation energy of HCHO in comparison to other VOCs such as ethanol (C_2_H_5_OH), methanol (CH_3_OH), acetone (C_3_H_6_O), isopropyl alcohol (IPA), and ammonia (NH_3_) (Han et al., 2022; Tang et al., 2022). Another possibility is reported that various gases have different lower unoccupied orbit energies (LUMO). In comparison to other VOCs, the electron energy of adsorbed oxygen species (E_ads_ = −0.35 eV/mol is nearly like the LUMO energy of HCHO (E_ads_ = −0.5 eV/mol), which facilitates a surface chemical reaction and hence the selectivity toward HCHO.

## Conclusions

In this study, Ni-Gr and WO_3_-Ni-Gr composites were synthesized by using a simple hydrothermal method for room-temperature formaldehyde gas sensing. SEM, TEM, XRD, RAMAN, XPS, and UV-Vis diffuse reflectance measurements were used to study the morphological, structural, and optical characteristics of both Ni-Gr and WO_3_-Ni-Gr composites. The characteristic results showed that walnut-like WO_3_ nanostructures with an average size of 53 ± 23 nm were successfully decorated on the surface of Ni-Gr. More oxygen vacancies O_v_ and the adsorbed oxygen species O_c_ on the surface of the WO_3_-Ni-Gr composite were generated in the WO_3_-Ni-Gr composite which enables the improved selectivity, high sensitivity at lower operating temperatures toward HCHO. The WO_3_-Ni-Gr sensor has shown a higher response of 86.8 against 500 ppm of HCHO in comparison to Ni-Gr with a response value of 22.7. In addition, the detection limit of the WO_3_-Ni-Gr sensor was reduced to 1 ppm while it was 10 ppm for the Ni-Gr composite. The sensing speed of the WO_3_-Ni-Gr-based sensor was fast for all concentrations in comparison to the Ni-Gr sensor. The improved selectivity and sensitivity of WO_3_-Ni-Gr can be attributed to the p-n heterojunction formation, rapid dissociation of oxygen molecules by both Ni and WO_3,_ and surface oxygen species which promotes the electron shuttling on HCHO interaction. The sensing performance of the WO_3_-Ni- Gr composite at different temperatures (25°C, 75°C, and 150°C) toward 50 ppm of HCHO has shown that sensors can be used at low and high temperatures for different applications. Excellent stability was also exhibited by the WO_3_-Ni-Gr based sensor toward 100 ppm of HCHO for 60 days making them promising candidates for designing an efficient device toward HCHO in practical applications.

## Data Availability

The original contributions presented in the study are included in the article/Supplementary Material; further inquiries can be directed to the corresponding author.
