# Supplementary figures and images for: A novel room-temperature formaldehyde gas sensor based on walnut-like WO3 modification on Ni–graphene composites
Source: Front Chem. 2022 Sep 9;10:971859. doi: 10.3389/fchem.2022.971859 (PMC9500379; doi:10.3389/fchem.2022.971859)

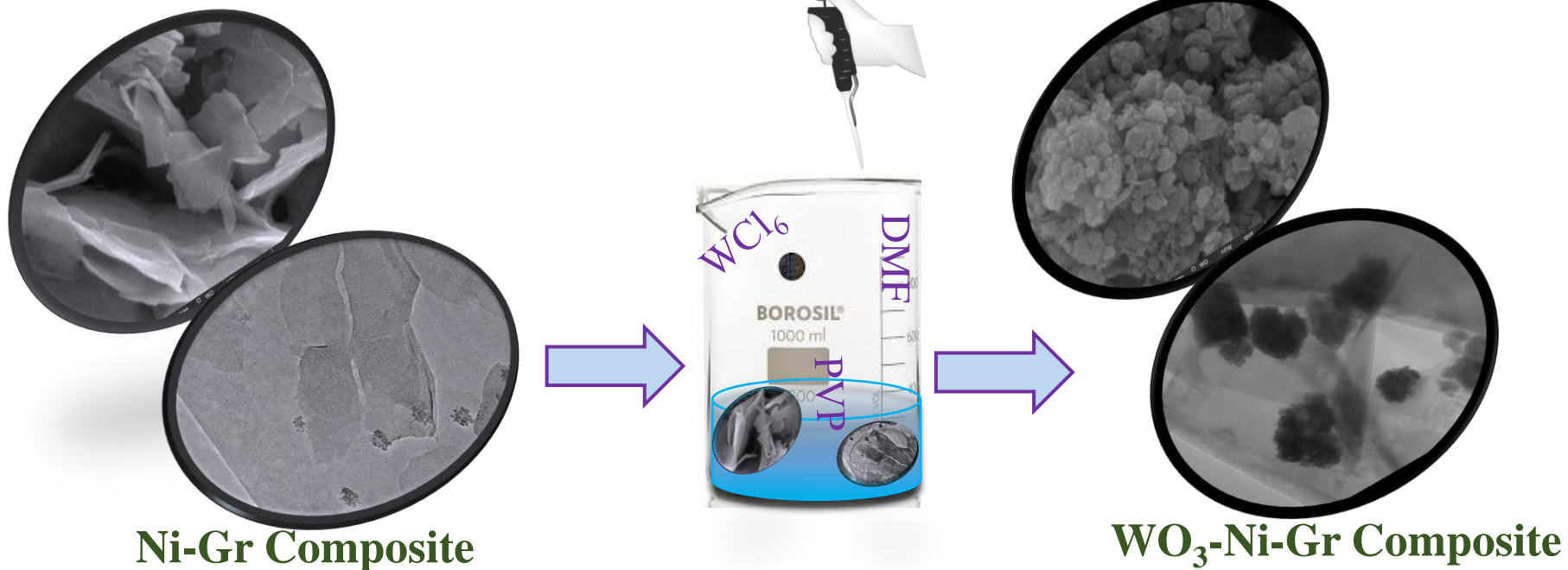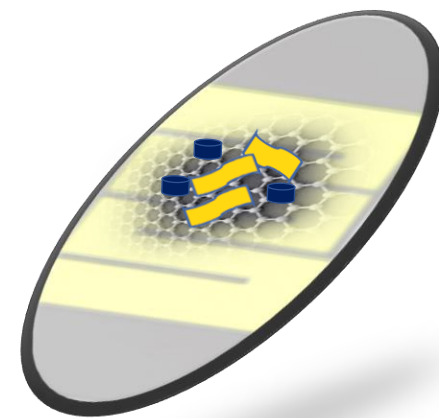

**Sensor device**

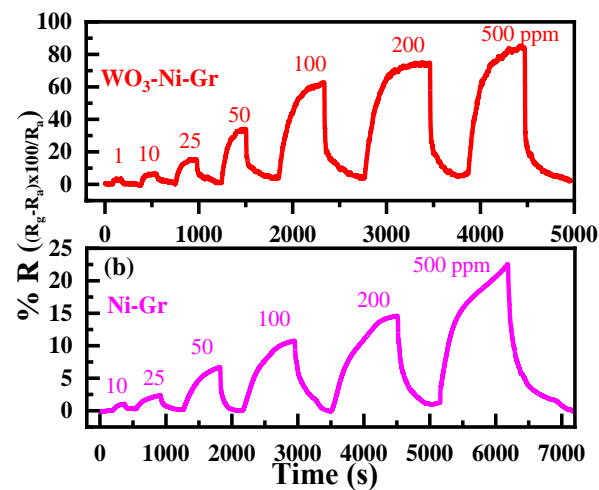

**Dynamic response**

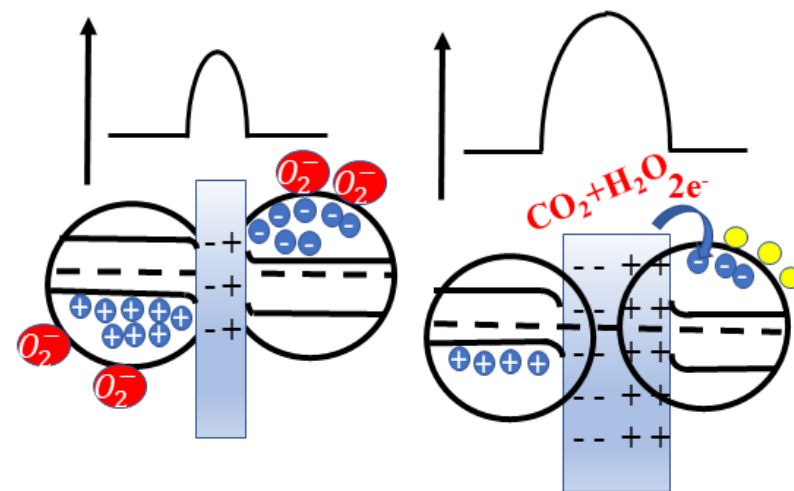

**In air**

**In HCHO**

**Graphical Abstract**

Supplement: Supplementary file 1 [file DataSheet2.PDF]
