# Supplementary material for: A novel room-temperature formaldehyde gas sensor based on walnut-like WO3 modification on Ni–graphene composites
Source: Front Chem. 2022 Sep 9;10:971859. doi: 10.3389/fchem.2022.971859 (PMC9500379; doi:10.3389/fchem.2022.971859)
Supplement: Supplementary file 2 [file DataSheet1.PDF]

A novel room-temperature formaldehyde gas sensor based on Walnut-like WO_3 modification on Ni-graphene composites

Shahid Mehmood[1], Faheem Ullah Khan[1], Muhmmad Naeem Shah[1], Ma Junxian[1], Yatao Yang[1], Guijun Li[2], Wei Xu[1], Xiaojin Zhao[1], Wei He[1], Xiaofang Pan[1,a]

Affliations:
[1]College of Electronics and Information Engineering, Shenzhen University, Shenzhen, Guangdong Province, P. R. China
[2]Key labortary of Optoelectronics Devices and System of Ministry of Education and Guangdong Province, College of Physics and Optoelctronics Engineering, Shenzhen University, Shenzhen, P.R. China
[a)] Author to whom correspondence can be addressed: eexpan@szu.edu.cn

Table of content

Figure S1: (a) Nyquist plots of WO_3, Gr, Ni-Gr composite, and WO_3-Ni-Gr composite. Inset shows the Randles equivalent circuits for determination of solution resistance (Rs), coating resistance (Rf), charge transfer resistance (Rct), double layer capacitance (Cdl) and a Warburg element (W). Mott-Schottky (MS) plot for (b) WO_3, (c) Ni-Gr composite, (d)WO_3-Ni-Gr composite. Red dotted line in each MS plot is used to approximate the value of intercept on potential axis for flat band potential Efb determination.

Table S1: Resistances (Rs, Rf and Rct) calculated from Nyquist plots by Randles equivalent circuit fitting in Z-view software

The electrochemical impedance spectroscopy (EIS) was performed to investigate the charge-transfer characteristics of WO_3, Gr, Ni-Gr and WO_3-Ni-Gr. The Nyquist plot for WO_3, Gr, Ni-Gr and WO_3 -Ni-Gr and the Randles equivalent circuit obtained by fitting in Z-view software is shown in Figure S1(a). EIS spectra fitted with Randles circuit have a semicircle at higher frequencies followed by a linear region. Randles model includes the solution resistance (Rs), coating resistance (Rf), charge transfer resistance (Rct), double layer capacitance (Cdl) and a Warburg element (W) to quantify the diffusion process in electrochemical systems. Corresponding values of Rs, Rf and Rct for WO_3, Gr, Ni-Gr and WO_3-Ni-Gr are given in Table S1. Table S1 indicated that the value of Rct for WO_3, Gr, Ni-Gr and WO_3-Ni-Gr are 700 Ω, 720 Ω, 400 Ω and 170 Ω, respectively. Value of Rct is the measure of charge transfer characteristics, smaller value of Rct reflects the enhanced charge transfer [39]. Lower value of Rct (170 Ω) for WO_3-Ni-Gr composite represented the enhanced electron transfer due to junction formation between WO_3 and Ni-Gr.

Table S1: Resistances (Rs, Rf and Rct) calculated from Nyquist plots by Randles equivalent circuit fitting in Z-view software.

Material	Rs (Ω)	Rf (Ω)	Rct (Ω)
WO_3	56	130	700
Gr	48	113	720
Ni-Gr	58	52	400
WO_3-Ni-Gr	44	32	170

Semiconductor type of the Ni-Gr composite, WO_3 nanostructures and WO_3-Ni-Gr composite was recorded by Mott-Schottky (MS) plot. MS measurements are carried out in 0 V to 1 V versus Ag/AgCl at a frequency of 10 KHz using three electrode system in 1M Na_2SO_4 electrolyte and is shown in Figure S1(b-d). From negative slope (Figure S1(b)) it was concluded that Ni-Gr shows a p-type behavior [40], positive slope (Figure S1(c)) of MS plot for WO_3 represents n-type behavior while negative slope [41] (Figure S1(d)) of WO_3-Ni-Gr composite confirmed the p-type characteristics. Flat band potential Efb which does not depend on time nor polarization of the layer can be determined by intercept of MS plot on potential axis [41]. Efb for Ni-Gr, WO_3 and WO_3-Ni-Gr are 0.64 V, -0.38 V and 0.75 V, respectively. In comparison to Ni-Gr, Efb is shifted to higher potential in WO_3-Ni-Gr composite, which indicated that the fermi level of p-type Ni-Gr is shifted upward while that of WO_3 is shifted

downward to form effective p-n junction formation [42]. Carrier density of the material is inversely proportional to the slope of the tangents in MS plots, and smaller the slope of tangent more will be the carrier density [43]. From Figure S1(b) and (d), the slope of the tangent is lower for WO_3-Ni-Gr composite in comparison to Ni-Gr composite and consequently carrier density will be high in WO_3-Ni-Gr composite. Enhanced carrier density for the ternary composite of WO_3-Ni-Gr is probably due to enhanced charge generation and efficient charge transfer and thus junction formation between WO_3 and Ni-Gr.

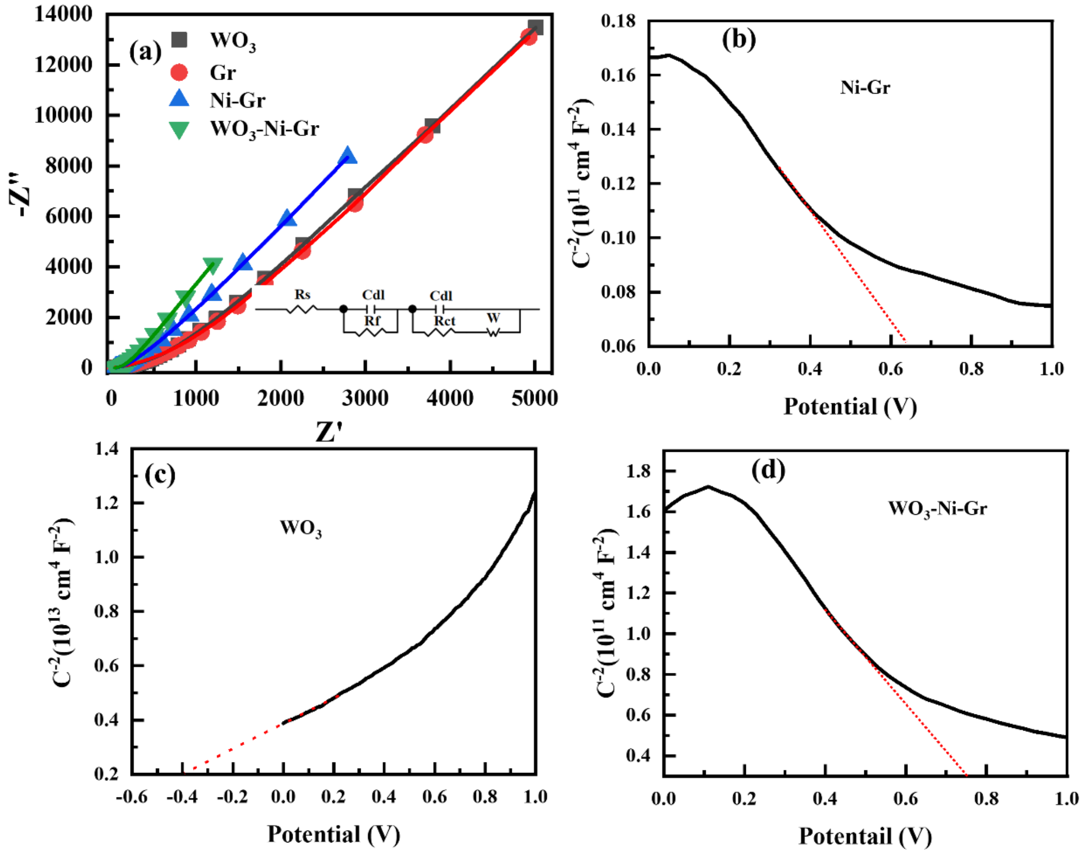

Figure S1: (a) Nyquist plots of WO_3, Gr, Ni-Gr composite, and WO_3-Ni-Gr composite. Inset shows the Randles equivalent circuits for determination of solution resistance (Rs), coating resistance (Rf), charge transfer resistance (Rct), double layer capacitance (Cdl) and a Warburg element (W). Mott-Schottky (MS) plot for (b) WO_3, (c) Ni-Gr composite, (d)WO_3-Ni-Gr composite. Red dotted line in each MS plot is used to approximate the value of intercept on potential axis for flat band potential Efb determination.
